# Effectiveness of esterified whey proteins fractions against Egyptian Lethal Avian Influenza A (H5N1)

**DOI:** 10.1186/1743-422X-7-330

**Published:** 2010-11-19

**Authors:** Soad H Taha, Mona A Mehrez, Mahmoud Z Sitohy, Abdel Gawad I Abou Dawood, Mahmoud M Abd-El Hamid, Walid H Kilany

**Affiliations:** 1 Dairy Science Department, Faculty of Agriculture, Cairo University, Egypt; 2 National Laboratory for Veterinary Quality Control on Poultry Production, Animal Health Institute, Ministry of Agriculture, Egypt; 3 Biochemistry Department, Faculty of Agriculture, Zagazig University, Zagazig, Egypt

## Abstract

**Background:**

Avian influenza A (H5N1) virus is one of the most important public health concerns worldwide. The antiviral activity of native and esterified whey proteins fractions (α- lactalbumin, β- lactoglobulin, and lactoferrin) was evaluated against A/chicken/Egypt/086Q-NLQP/2008 HPAI (H5N1) strain of clade 2.2.1 (for multiplicity of infection (1 MOI) after 72 h of incubation at 37°C in the presence of 5% CO_2_) using MDCK cell lines.

**Result:**

Both the native and esterified lactoferrin seem to be the most active antiviral protein among the tested samples, followed by β- lactoglobulin. α-Lactalbumin had less antiviral activity even after esterification.

**Conclusion:**

Esterification of whey proteins fractions especially lactoferrin and β-lactoglobulin enhanced their antiviral activity against H5N1 in a concentration dependent manner.

## Background

Avian influenza A (H5N1) virus is one of the most important public health concerns worldwide. It has been detected and identified in South East Asia since 2003. In 2005, the virus was already spread to many countries in Europe, Asia and Africa [1]. Later it interrupted international travel and negatively affected the world economy especially tourism.

In December 2005, the first case of H5N1 in Egypt was detected in a migrating bird in Damietta Governorate. In mid-February 2006, H5N1 infection was reported in Egypt among domestic poultry in more than 15 governorates, resulting in severe losses for the poultry industry. In March 2006, the first human case of H5N1 in Egypt was detected [2]. Egypt had the highest number of confirmed human avian influenza cases outside Asia. As of 4 March 2010, 104 human cases, including 30 fatalities, had been recorded in Egypt [3,4].

Whey proteins have been reported to have numerous therapeutic applications including effects on bone (stimulate proliferation and differentiation of osteoblastic cells as well as suppress bone resorption and increase femoral bone strength), muscles (enhancing muscle hypertrophy and strength due to its leucine content), blood (lower blood pressure and reduce the risk of hypertension), brain (increase brain serotonin levels), immune system (stimulate immunity and improving immune function), cancer (increase NK cell function and glutathione levels), wound healing (essential for post-surgical wound healing and protein depletion delays healing time), and aging (antiaging agent due to the whey content of glutathione/antioxidant component). Furthermore, they act as antioxidant, antihypertensive, antiviral, antimicrobial, chelating agent and prevent cardiovascular diseases and osteoporosis [5,6]. The biological value of whey proteins has also been reviewed for their antimicrobial and antiviral functions [7-11].

Modification of whey proteins to enhance or alter their biological and functional properties may increase its applications. Whey proteins modification can be accomplished by chemical, enzymatic, or physical techniques [12]. Additional negative charges on β-lactoglobulin (BLG), α-lactalbumin (ALA) and human serum albumin (HSA) endowed them a significant antiviral activities against human immunedeficiency viruses—HIV-1 and HIV-2 [13-17]. BLG modified with 3-hydroxyphthaloylacid (3HP) inhibited the infection with HIV-1, Herpes simplex virus types 1 and 2 and human cytomegalovirus [18,19]. In addition, many studies reported that increasing the net positive charge on whey proteins led to enhancement of its antiviral activity. Esterified whey proteins showed antiviral activity against poliovirus type-1, Coxsackie virus B6, human cytomegalovirus, Herpes simplex virus type 1 and human influenza virus A subtype H3N2 & subtype H1N1 [20-24].

Therefore, it was thought worthwhile to test the efficacy of native and modified whey proteins fractions against influenza virus subtype H5N1which may be useful for the prophylaxis and treatment of influenza viruses and at the same time can be a potential and low cost alternative candidate for an anti-influenza agent.

## Materials and methods

### Materials

The HPAI H5N1 A/chicken/Egypt/086Q-NLQP/2008 (referred to as EGYvar/H5N1) virus (GenBank accession number: EU496398.1) was isolated from an H5 vaccinated commercial chicken farm in 2008 during the routine national surveillance conducted by the National Laboratory for Veterinary Quality Control on Poultry Production (NLQP), Giza, Egypt. The virus has been titrated using Hemagglutination test (HA) 256 HAU. The tissue culture lethal dose 100% (TCLD100%), tissue culture infective dose 50% (TCID50%) and Embryonated egg infected dose 50% (EID50%) were calculated using Read and Minch (10^6^, 10^8.26^, 10^8.64^/ml respectively) in Specific Pathogen Free Embryonated Chicken Egg (SPF ECE) and Madin-Darby Canine Kidney Cells (MDCK) as described in WHO manual [25]. SPF ECE was obtained from Qom Oshime SPF farm Egypt, and MDCK was obtained from NAMRU3 unit, Egypt.

α-Lactalbumin (97.46% protein) and β-lactoglobulin (97.8% protein) were kindly obtained from Davisco Food International (USA) while lactoferrin (95% protein) was kindly obtained from Armor Proteins (France). All other chemicals used in this study were of analytical grade.

### Methods

#### 1-Protein Esterification

The procedure of [26] was used for esterification of whey proteins fractions.

#### 2-Esterification extent

The extent of esterification of proteins was quantified by the colour reaction with hydroxylamine hydrochloride as described by [27].

#### 3- The antiviral activity of native and esterified whey protein

The antiviral activity of native and esterified whey proteins: α-lactalbumin (ALA), β-lactoglobulin (BLG) and lactoferrin (LF) was assayed against Egyptian highly pathogenic avian virus A/chicken/Egypt/086Q-NLQP/2008 HPAI (H5N1) strain of clade 2.2.1 at concentrations of 1.00 MOI (multiplicity of infection) per cell according to [22].

#### 4- Statistical analysis

All experiments were performed in triplicates and the results were expressed by the mean plus the standard deviation.

## Results

### Extent of esterification

The whey proteins fractions ALA, BLG and LF were modified to the extent of 68%, 100% and 100% respectively which indicates less esterification susceptibility of ALA as compared to both BLG and LF. The observed extents of such esterification are in accordance with [26].

### Antiviral activity of whey proteins fractions

**Figure 1:**
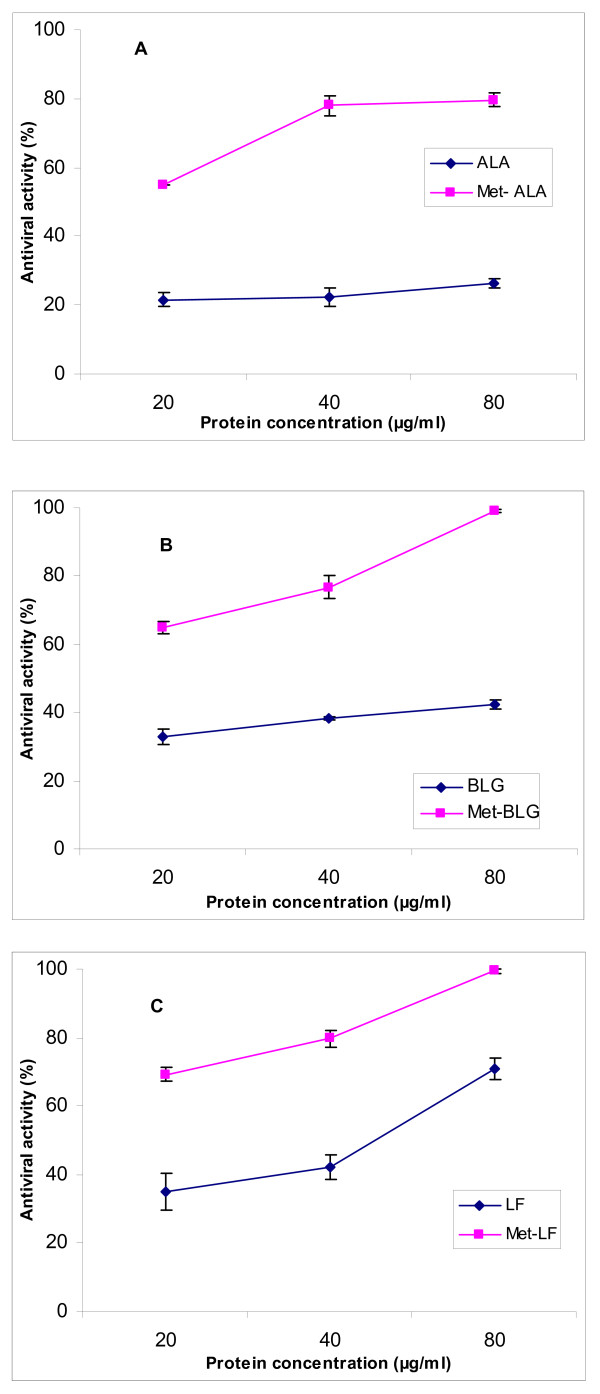
**Antiviral activity of native and esterified whey proteins fractions against H5N1 virus infecting MDCK cells**. A: α-lactalbumin, B: β-lactoglobulin and C: lactoferrin.

Data shown in Figure 1 demonstrate the antiviral effect of native and esterified whey proteins fractions against H5N1 propagated in MDCK cells at 100% (1.00 MOI) level of viral infection. Native proteins have exhibited different levels of inhibitory effects against the virus. It ranged from 21.62 ± 2.1 to 26.40 ± 1.5% for ALA, from 32.87 ± 2.3 to 42.43 ± 1.3% for BLG and from 34.98 ± 5.5 to 70.92 ± 3.2% for LF in response to protein concentration increasing from 20 to 80 μg/ml. The difference in viral inhibitory effect of the three native proteins may be due to the difference in their structural nature. Native lactoferrin seems to be the most active antiviral protein among the tested samples, probably due to its more basic nature that enables its interference and interaction with the viral constituents affecting viral replication and activity. Esterification of whey proteins fractions has further enhanced their antiviral activity against H5N1 in a concentration dependent manner. Met-ALA exhibited antiviral effect ranging from 54.84 ± 0.1 to 79.57 ± 2.0%, Met-BLG from 64.88 ± 1.9 to 99.05 ± 0.4% and Met-LF from 69.28 ± 1.8 to 99.42 ± 0.6% in response to protein concentration going from 20 to 80 μg/ml. Met-ALA was the lowest active as antiviral protein even after esterification while both Met-BLG and Met-LF reached maximum antiviral influence when the protein concentration was increased to 80 μg/ml. This may confirm that esterification is a potent tool which introduces this antiviral activity into native proteins. It may also show that the original differences between BLG and lactoferrin disappeared completely after esterification.

The antiviral activity of the tested proteins may be due to its interaction with influenza nuclear proteins (PB1, PB2, PA and NP), which catalyze the transcription of viral RNA [28-30]. Since these proteins are normally associated with RNA and undergo systematic dissociation during replication, the tested positively charged proteins may interfere with this association-dissociation process or compete for the negative charges on the exposed regions of RNA, disturbing the overall replication pathways.

## Conclusion

From the presented data, it can be concluded that esterification of LF followed by BLG and lastly by ALA enhances its antiviral activities against H5N1 infected into MDCK cell lines which is dependent on the concentration of the tested proteins. Consequently, applying this technique is associated with a protective action on the cell lines subjected to the viral infection. Further studies are needed to improve the antiviral activity of both of α-lactalbumin and to a less extent β-lactoglobulin.

## Competing interests

The authors declare that they have no competing interests.

## Authors' contributions

SHT carried out the study design, participated in data organization, wrote and revised the manuscript; MAM helped in performance of the experiments; MZS carried out the study design and revised the manuscript; AIA participated in the design of the study and revised the manuscript; MMA carried out most of the experiments, participated in data organization, wrote and revised the manuscript; WHE helped in performance of the experiments.

All authors read and approved the final manuscript.
